# 3D-cultivation of NSCLC cell lines induce gene expression alterations of key cancer-associated pathways and mimic *in-vivo* conditions

**DOI:** 10.18632/oncotarget.22636

**Published:** 2017-11-06

**Authors:** Gabriele Gamerith, Johannes Rainer, Julia M. Huber, Hubert Hackl, Zlatko Trajanoski, Stefan Koeck, Edith Lorenz, Johann Kern, Reinhard Kofler, Jens M. Kelm, Heinz Zwierzina, Arno Amann

**Affiliations:** ^1^ Medical University of Innsbruck, Department of Internal Medicine V, 6020 Innsbruck, Austria; ^2^ Tyrolean Cancer Research Institute, 6020 Innsbruck, Austria; ^3^ Medical University of Innsbruck, Biocenter, Division of Molecular Pathophysiology, 6020 Innsbruck, Austria; ^4^ European Academy of Bolzano/Bozen (EURAC), Center for Biomedicine, 39100 Bolzano, Italy; ^5^ Medical University of Innsbruck, Biocenter, Division of Bioinformatics, 6020 Innsbruck, Austria; ^6^ Oncotyrol, Innsbruck, 6020 Innsbruck, Austria; ^7^ InSphero AG, 8952 Schlieren, Switzerland

**Keywords:** 3D cell culture, in-vivo, gene expression, pathways in carcinogenesis, lung cancer

## Abstract

This work evaluated gene expression differences between a hanging-drop 3D NSCLC model and 2D cell cultures and their *in-vivo* relevance by comparison to patient-derived data from The Cancer Genome Atlas.

Gene expression of 2D and 3D cultures for Colo699 and A549 were assessed using Affymetrix HuGene 1.0 ST gene chips. Biostatistical analyses tested for reproducibility, comparability and significant differences in gene expression profiles between cell lines, experiments and culture methods.

The analyses revealed a high interassay correlation within specific culture systems proving a high validity. 979 genes were altered in A549 and 1106 in Colo699 cells due to 3D cultivation. The overlap of changed genes between the cell lines was small (149), but the involved pathways in the reactome and GO- analyses showed a high overlap with DNA methylation, cell cycle, SIRT1, PKN1 pathway, DNA repair and oxidative stress as well known cancer-associated representatives. Additional specific GSEA-analyses revealed changes in immunologic and endothelial cell proliferation pathways, whereas hypoxic, EMT and angiogenic pathways were downregulated.

Gene enrichment analyses showed 3D-induced gene up-regulations in the cell lines 38 to be represented in *in-vivo* samples of NSCLC patients using data of The Cancer Genome Atlas.

Thus, our 3D NSCLC model might provide a tool for early drug development and investigation of microenvironment-associated mechanisms. However, this work also highlights the need for further individualization and model adaption to address remaining challenges.

## INTRODUCTION

Within recent years drug development was hampered by the high failure rate of substances in clinical trials [1–3]. This is partially caused by the lack of sufficient predictive preclinical models [4]. Therefore, there is a unmet need for biomarker research [3] and a major effort towards more relevant drug testing systems [5, 6].

Traditional monolayer cultures do not sufficiently reflect targeted drug responses at tissue levels [7] with various differing mechanisms, such as immunological process, epithelial mesenchymal transition (EMT), hypoxia and stemness [8]. Therefore, microtissues gained attention with their nutrient and oxygen gradients and microenvironmental factors including cell-to-cell interactions [9–12], thereby reflecting *in-vivo* tumor growth more reliably. Besides, their human origin, better cost effectiveness compared to animal models and reduced ethical issues favor this approach.

Hence, several 3D culture systems have been established, including scaffolds, matrix gels and hanging drops, each with distinct advantages and limitations [5, 12, 13].

The aim of our work was to highlight these differences in gene expression patterns between 2D and 3D for NSCLC cell lines, their comparability, role in cancer-associated pathways and *in-vivo* relevance.

Previous investigations stated relevant differences in gene expression profiles for several other cell lines cultured by different techniques, mainly scaffolds and matrix gels [11, 14–16].

For our work, the hanging drop model fulfilled our requirements to minimize external factors, such as the addition of growth factors and external matrices on the one hand, and to enable long-term cultivation and drug addition on the other.

We tested two non-small-cell lung cancer cell lines, Colo699 and A549, for their differences in the mRNA expression profiles cultured in 2D or 3D with our prior established hanging drop lung cancer model [8].

## RESULTS

We generated A549 and Colo699 microtissues using the hanging drop technology. The 2D cultured cells were harvested after 5 days, whereas 3D microtissues of Colo699 were harvested after 10 days and A549 after 5 and 10 days, respectively.

**Figure 1:**
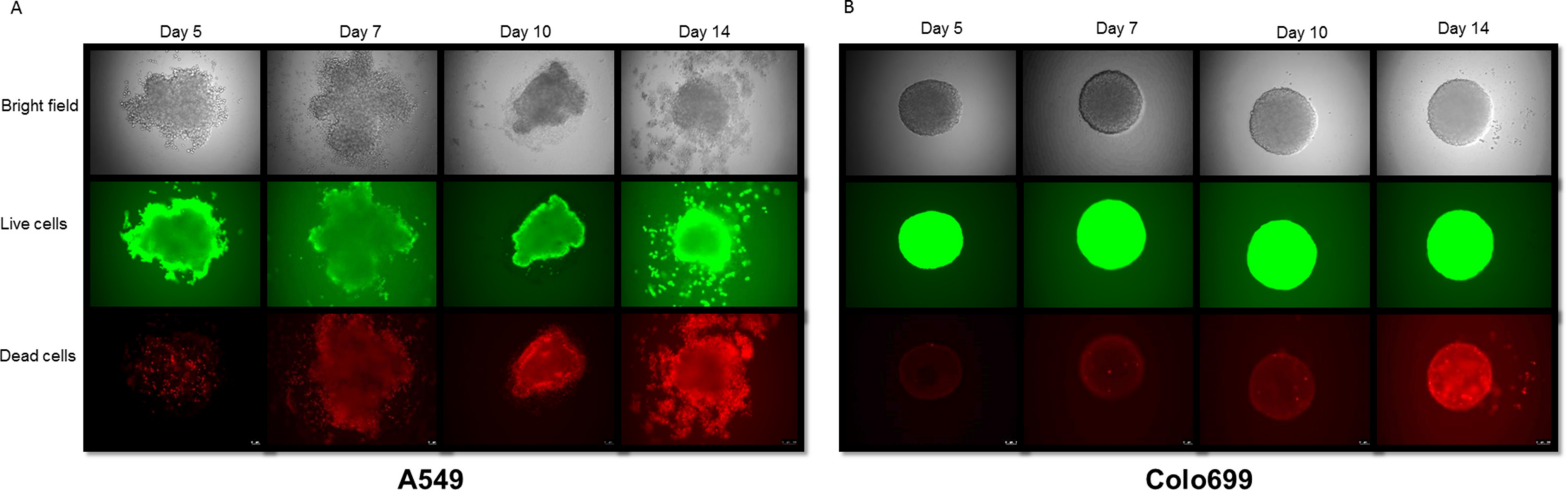
Morphological and viability assessment of spheroids Bright field microscopy and calcein AM (live cells) and ethidium homodimer-1 (dead cells) fluorescence staining of 3D spheroids of A549 cells (**A**; left) and Colo699 cells (**B**; right) show the most stable spheroid structure with a limited number of dead cells at 10 days of cultivation.

The ten days of cultivation for 3D cultures were chosen based on our prior experience concerning microspheroid aggregation [8]. This was further evaluated and proven by light microscopic appearance, as well as viability tests for 3D cultures after 5, 7, 10 and 14 days by direct fluorescence staining and microscopic evaluation, as depicted in Figure 1A and 1B.

Cells were harvested, pooled and RNA isolation was performed for all cell lines and culture techniques followed by quality assessment, as indicated methods section. The chip analyses were run and raw data were processed following the procedures described within the statistical methods part.

**Figure 2:**
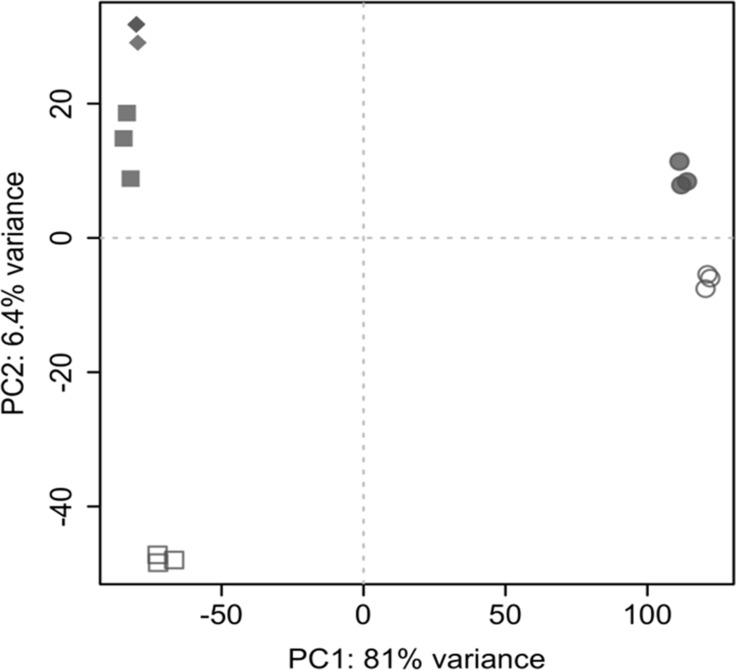
Principal component analysis Separation occurs between cell lines (Colo699: circles; A549: rectangles) and culture conditions (2D culture condition: open symbols; 3D: filled symbols). 2D cultures were cultivated for 5 days, whereas 3D cultures were harvested after 10 days with the exception of the samples indicated by diamonds, which represent A549 cells cultured in 3D for 5 days.

A principal component analysis performed on the whole genome gene expression profiles revealed a clear separation of cell lines on principal component 1 (PC1) and culture techniques on principal component 2 (PC2) (see Figure 2).

The observable low interreplicate variability along with the above described differences between sample groups demonstrate robust data and the results confirm differences in gene expression profiles between culture techniques and cell lines.

### Gene expression changes induced by cultivation technique

We next compared the gene expression profiles of cells cultivated in 3D cell cultures with those from cells cultivated in conventional 2D cultures. Thereby, 979 and 1106 genes were identified to differ significantly in A549 and Colo699 cells, respectively (see Table 1 for the 30 most significantly altered genes in each cell line).

**Table 1:** 30 most significant gene regulations

A549 3D vs 2D culture			Colo699 3D vs 2D culture		
Gene	FDR	log2FC	Gene	FDR	log2FC
CEACAM5	< 0.001	6,60	ADCY8	< 0.001	4,30
BPIFB1	< 0.001	6,30	CLEC1A	< 0.001	4,10
BPIFA1	< 0.001	6,10	C12orf39	< 0.001	3,70
CATSPERB	< 0.001	5,50	TENM2	< 0.001	3,50
FCGBP	< 0.001	5,30	LINC00277	< 0.001	3,40
F5	< 0.001	4,90	RIMS1	< 0.001	3,40
TCN1	< 0.001	4,80	RP11–809H16.5	< 0.001	3,40
TSPAN1	< 0.001	4,60	TPD52L1	< 0.001	3,30
CEACAM6	< 0.001	4,50	METTL7A	< 0.001	3,20
NOX1	< 0.001	4,50	ST8SIA1	< 0.001	3,10
ADH6	< 0.001	4,40	OCLN	< 0.001	3,10
UGT2B15	< 0.001	4,30	SLC6A20	< 0.001	3,10
LYZ	< 0.001	4,30	CD96	< 0.001	3,10
AQP3	< 0.001	4,20	MRGPRX3	< 0.001	3,00
CST1	< 0.001	4,10	ASS1	< 0.001	3,00
AGR3	< 0.001	4,00	COLEC12	< 0.001	2,90
GRHL3	< 0.001	4,00	IGF1	< 0.001	−3,80
LAMA4	< 0.001	3,80	MAP7D2	< 0.001	−3,60
FER1L6	< 0.001	3,80	OR4A16	< 0.001	−3,50
KCNJ2	< 0.001	3,70	GPR56	< 0.001	−3,50
ITIH2	< 0.001	3,70	SPP1	< 0.001	−3,50
C9orf152	< 0.001	3,60	MLPH	< 0.001	−3,40
TM4SF4	< 0.001	3,60	U1	< 0.001	−3,20
GDA	< 0.001	3,50	STON2	< 0.001	−3,10
IL33	< 0.001	3,50	PCDH17	< 0.001	−3,00
CFB	< 0.001	3,50	GALNT3	< 0.001	−3,00
CDCP1	< 0.001	−3,20	ARHGAP28	< 0.001	−3,00
GREM1	< 0.001	−3,50	LMO3	< 0.001	−2,90
FAM129A	< 0.001	−3,50	SULF1	< 0.001	−2,90
LOXL2	< 0.001	−4,20	CMKLR1	< 0.001	−2,90

The table lists the 30 most affected genes by 3D versus 2D cell cultivation for A549 and Colo699 (red: up-regulated, blue: down-regulated)

FDR is false discovery rate as used for the multiple testing correction of *p*-values according to the Benjamini Hochberg method; log2FC is log2 fold change.

**Figure 3:**
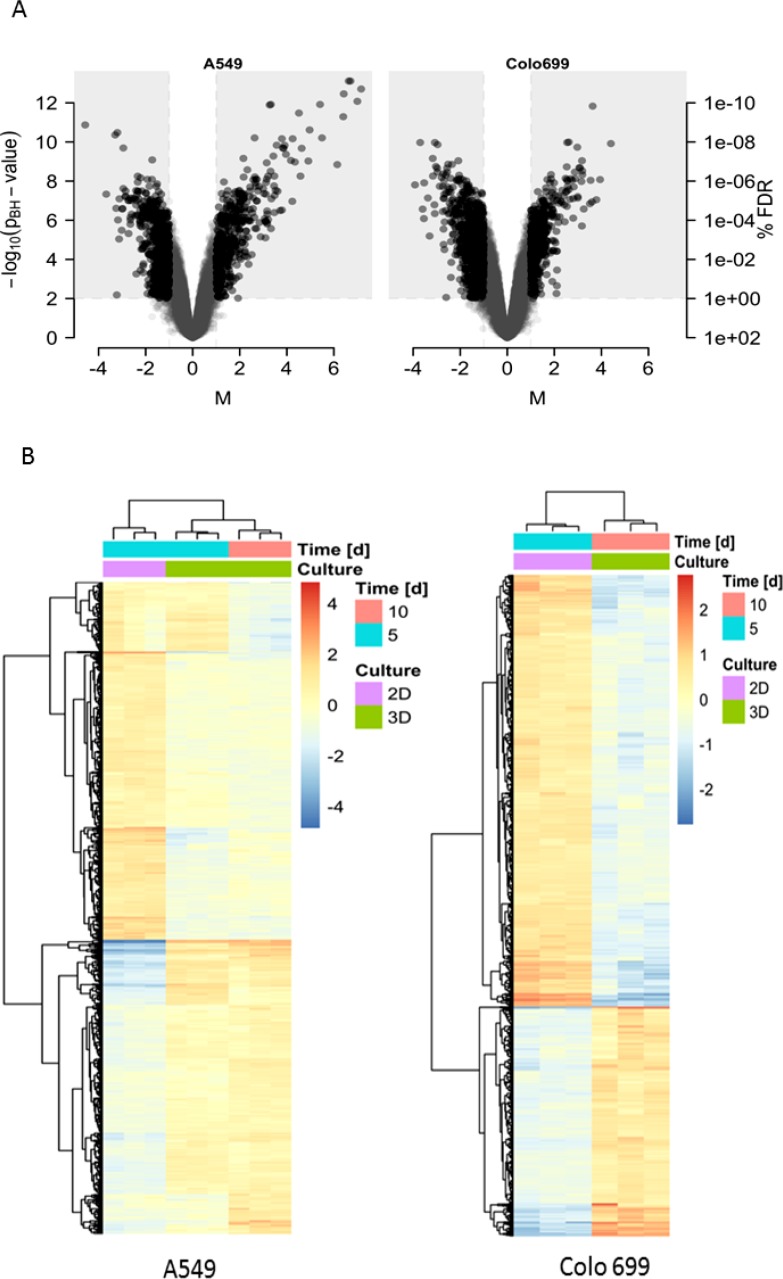
Gene expression differences (**A**) The Volcano plots represent the differences in gene expression between 3D and 2D cell culture conditions for A549 (left) and Colo699 cells (right). The extent of the expression alterations are shown on the x-axis (M or log2 fold change value) against its significance on the y-axis (-log10 of the adjusted *p*-values). Genes in the light-grey rectangular regions are those defined as significantly altered. (**B**) The heat maps depicture differentially expressed gene profiles between 2D and 3D cultures for A549 (left) (2D cultures (pink) after 5 days (blue) and 3D cultures (green) after 5 (blue) & 10 (red) days) and Colo699 (right) (2D cultures (pink) after 5 days (blue) and 3D cultures (green) after 10 days (red).

The expression differences were highlighted in the volcano plots (Figure 3A) and the heatmaps for both cell lines (Figure 3B).

Only a small overlap of regulated genes (149 genes in total) was identified between both cell lines (see Table 2 for a list of the most relevant changes in both cell lines and Supplementary Figure 1), suggesting that these two cell lines respond differently to the 3D cell culture condition. To rule out a potential cut-off bias [18], we directly compared the responses to 3D cultivation of both cell lines. The large number of differentially regulated genes in this comparison (1672) confirmed the findings above. Thus, on the individual gene level, the responses differed considerably between the two cell lines.

**Table 2:** Concordant regulated genes for both cell lines

	Gene expression changes 3D vs 2D			
	A549		Colo699	
Gene	FDR	log2FC	FDR	log2FC
EFCAB4B	< 0.001	2,70	< 0.001	1,20
PDE3A	< 0.001	2,20	< 0.001	1,80
**UNC13A**	< 0.001	2,20	< 0.001	2,10
CEACAM1	< 0.001	1,30	< 0.001	2,50
PKDCC	< 0.001	1,20	< 0.001	2,40
**AZGP1**	< 0.001	2,00	< 0.001	2,10
FAM129A	< 0.001	−3,50	< 0.001	−1,50
RBM24	< 0.001	−2,70	< 0.001	−1,40
ZEB2	< 0.001	−2,50	< 0.001	−1,30
FOXM1	< 0.001	−2,30	< 0.001	−1,50
CDC20	< 0.001	−2,10	< 0.001	−1,70
FHOD3	< 0.001	−2,10	< 0.001	−1,90
JAG1	< 0.001	−2,10	< 0.001	−1,80
**TNS1**	< 0.001	−2,10	< 0.001	−2,60
CENPI	< 0.001	−1,60	< 0.001	−2,50
GPNMB	< 0.001	−2,00	< 0.001	−2,10
HAS2	< 0.001	−1,60	< 0.001	−2,00
GRIN2B	< 0.001	−1,50	< 0.001	−2,00

The table describes the concordant altered genes in both cell lines (A549 and Colo699) with at least a 2-fold change in expression within one cell line.

FDR is false discovery rate as used for the multiple testing correction of *p*-values according to the Benjamini Hochberg method; log2FC is log2 fold change.

**Table 3:** Summary of validation qPCR results

Microarray			2D/3D day 10			
			A549		Colo699	
Cell line	log2 FC		log2 FC	Stabw	log2 FC	Stabw
A549	4,9	F5	3,2	0,2	1,2	0,4
A549	3,6	TM4SF4	2,8	0,4	0,4	0,5
A549	−3,1	CA9	−3,8	0,1	−0,5	0,1
Colo699	−3,8	IGF	0,7	0,2	−2,8	0,1

The table summarizes the results of the validation qPCRs, depicting the log2 ΔΔCT values for each cell line (right) in comparison to the original microarray data (left).

### Validation of multiarray data by RT-PCR

**Figure 4:**
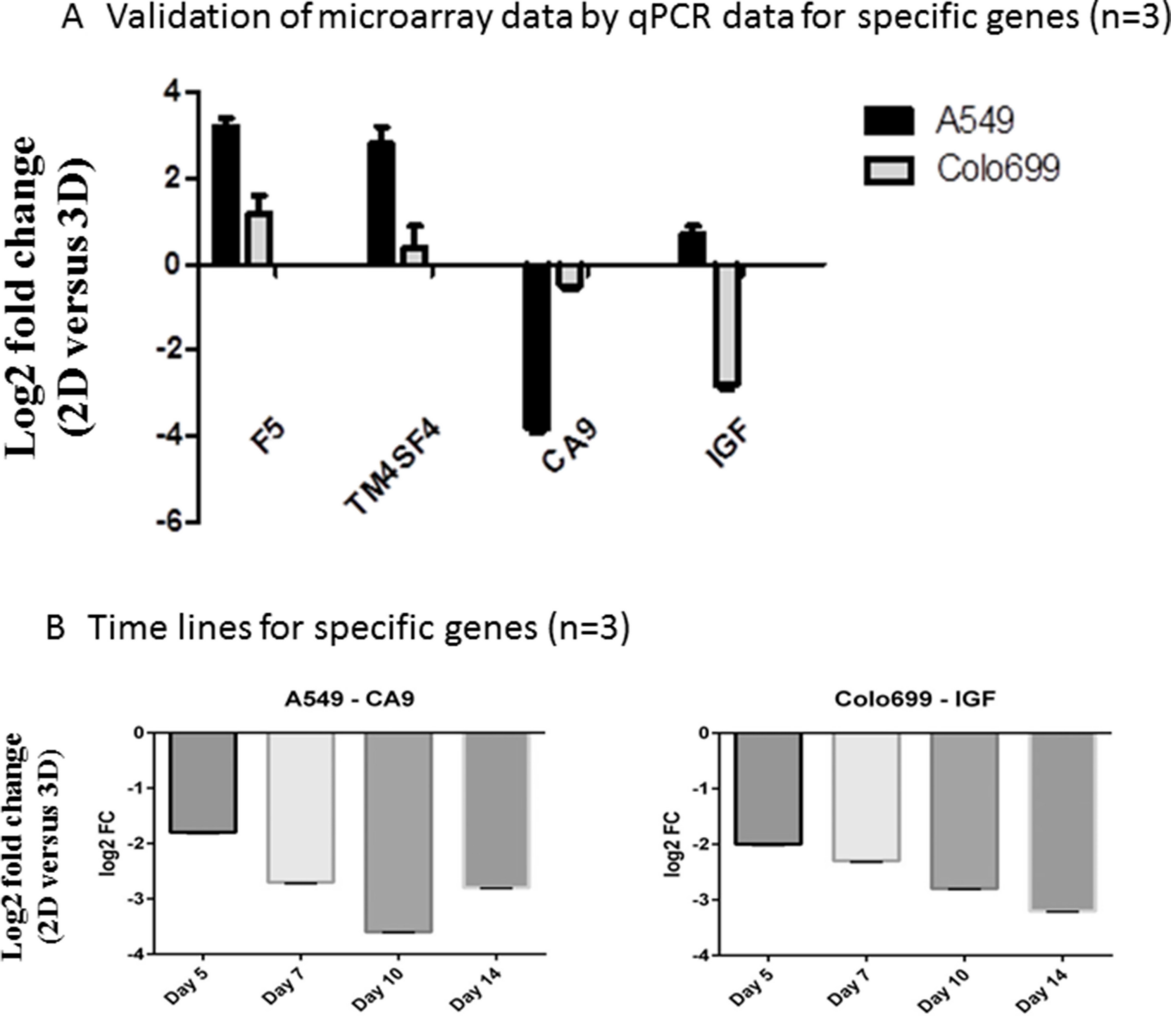
Validation of microarrays by qPCR Specific up- and down-regulated genes between 2D and 3D were measured by qPCR in A549 and Colo699 cell lines (**A**) and time series for the down-regulated genes in A549 and Colo699 (**B**) were run to provide additional insights in gene expression changes due to cultivation time and underline the chosen time points for the microarrays (*n* = 3; error bars depict SD).

To validate the multi-array findings, we ran additional qPCR tests for specific genes, either up- or down-regulated in one of the cell lines. We confirmed up-regulation of F5 and TM4SF4 and down-regulation of CA9 in the A549 cell line. Additionally, down-regulation of IGF1 was confirmed in the Colo699 cell line (see Figure 4 and Table 4). These data show reproducibility of our results.

**Table 4:** Sequences of primer pairs for qPCR analysis

Gene name	Forward primer	Reverse primer
IGF1	5′-ATGTGACATTGCTCTCAACA-3′	5′-GCATCTTCACCTTCAAGAAATC-3′
CA9	5′-TTTGAATGGGCGAGTGATT-3′	5′-AGGAATTCAGCTGGACTGG-3′
F5	5′-GACATCGCCTCTGGGCTAAT-3′	5′-GATGTCTGCTGCCCTCTGTA-3′
TM4SF4	5′-GCGATTTGCGATGTTCACCT-3′	5′-AAGGGGTAGCCCCATGTACT-3′
18SRNA^*^	5′-GTTGGTGGAGCGATTTGTCT-3′	5′-GGCCTCACTAAACCATCCAA-3′

^*^House keeping gene.

In an additional analysis, we investigated the time-dependent regulation of CA9 and IGF1 after 5, 7, 10 and 14 days of cultivation in 3D. These data underline the morphological and viability assessments, as they suggest a stabilization of the spheroids after 10 days (Figure 4B).

### Pathway and GO enrichment analyses

**Figure 5:**
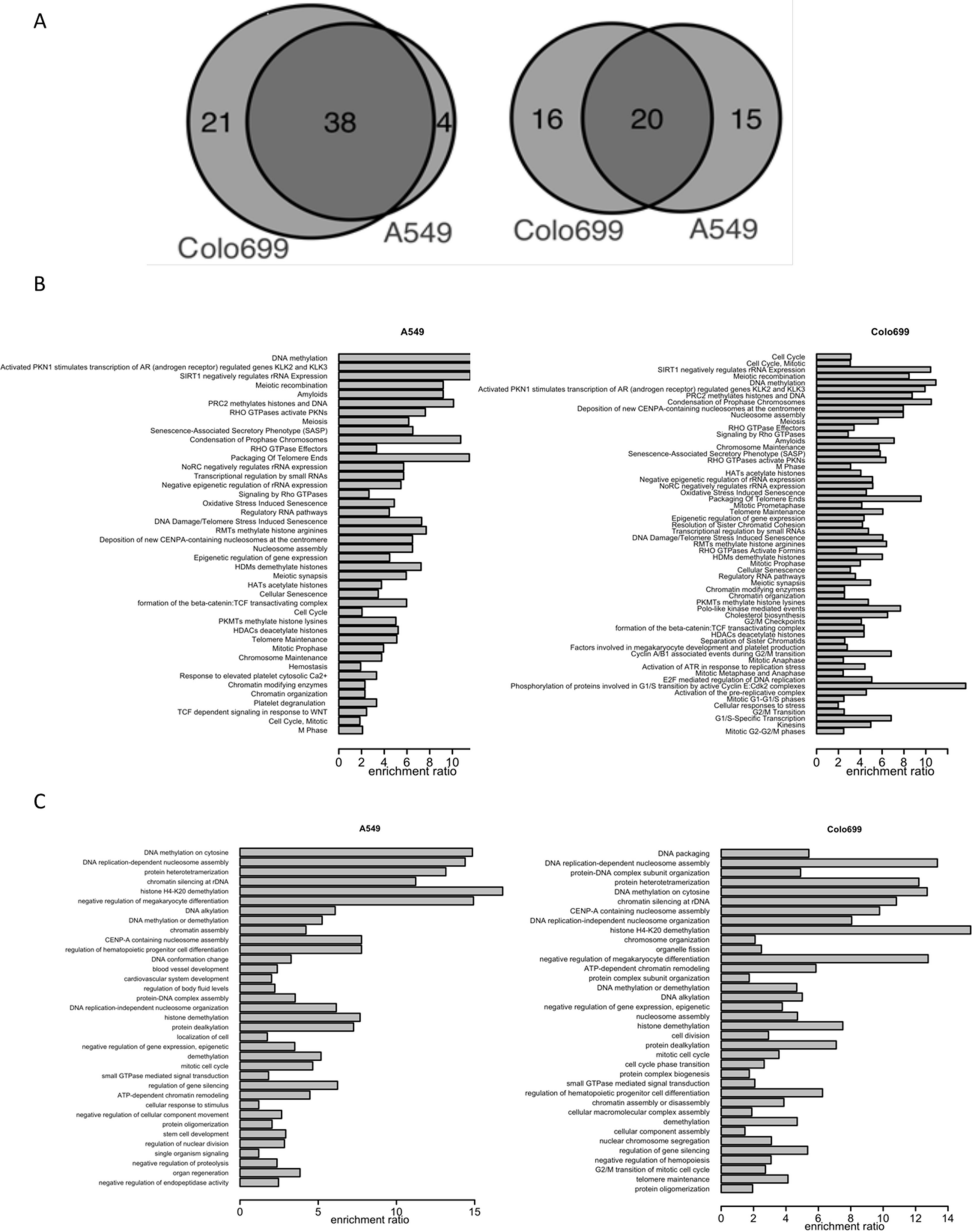
Overlap and enrichment analyses of altered reactome and G0 biological pathways (**A**) The venn diagrams represent the number of significantly enriched reactome pathways (left) and GO biological pathways (right) between 3D and 2D cell cultures for A549 and Colo699 cells. (**B** and **C**) The enrichment analyses summarize the differentially expressed genes between 2D and 3D cultures according to reactome pathways (B) and GO biological pathways (C) for A549 (left) and Colo699 (right) cell lines, respectively.

Next we performed Reactome Pathway and Gene Ontology (GO) Enrichment Analyses to identify the affected biological processes and pathways. We found 42 and 59 reactome (Figure 5B; Supplementary Tables 2 and 3) and 35 and 36 biological pathways (Figure 5C; Supplementary Tables 4 and 5) with significant enrichment in A549 and Colo699 cells, respectively.

For both cell lines most of the genes were involved in DNA methylation and packaging, cell cycle, SIRT1, PKN1 pathways, as well as DNA repair and oxidative stress pathways (Figure 5). Although the overlap of differentially expressed genes was small, the overlap of enriched pathways and, to a lower extend, of biological processes was strikingly high (Figure 5A). The different culture conditions induced comparable changes in gene expression profiles for data aggregated over reactome or biological pathways in both cancer cell lines.

The 10 day microtissue culture time was chosen based on our prior experiments [8], where spheroid aggregation was observed to be most stable after 10 days (Figure 1 and 4B). As some of the gene expression changes might be attributed to the different culture times prior harvesting (i.e. 5 days for 2D and 10 days for 3D culture), we additionally compared gene expression profiles of A549 cells cultivated for 5 days in 3D cultures with the corresponding 2D and 10 day 3D cultures. Thus, 1321 genes were identified to be differentially expressed between 5 day cultivation in 2D and 3D, about half (632) of them were also differentially expressed between 10 day 3D and 5 day 2D cultures. Although more gene expressions were altered after 5 days of 3D culture, these were significantly overrepresented in fewer biological pathways (15 compared to 42) and biological processes (15 compared to 35) (Supplementary Tables 5–6). All of the enriched pathways and 66.6% of biological processes were also enriched in the comparison between 10 day 3D and 5 day 2D cultures (Supplementary Figure 2). These results, in combination with our morphological assessments and viability measurements (Figure 1), as well as the qPCR data (Figure 4B) support that 3D cultures remain viable and adapt to culture conditions after 10 days, whereas after 5 days this adaption was still ongoing. This was reflected by the larger perturbation in the gene expression profiles but lesser involved biological processes.

### Specific gene signature enrichment analyses (GSEA)

**Figure 6:**
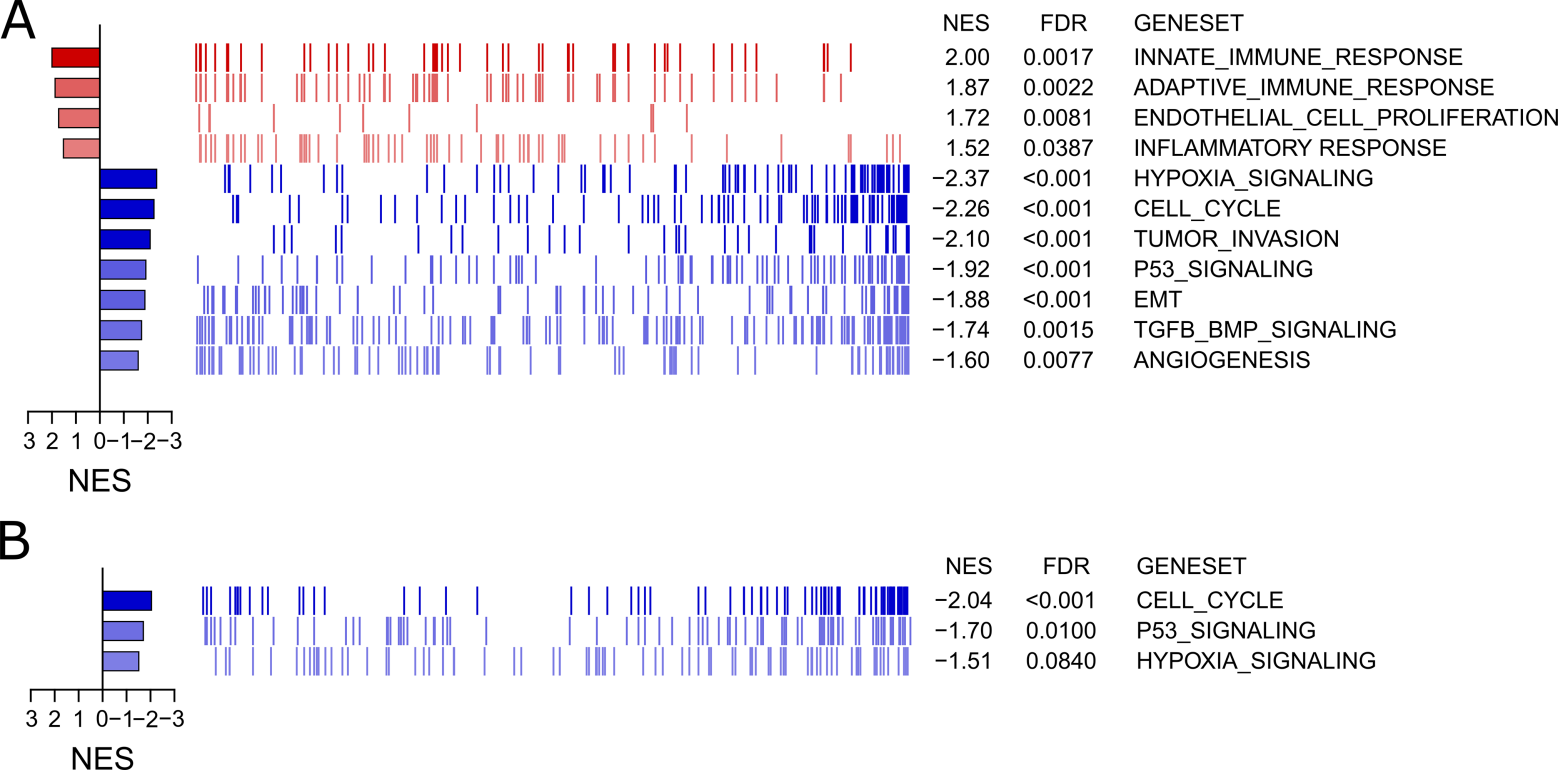
Gene signature enrichment analyses for specific pathways The GEAs depict the enrichment of altered genes for specific, chosen hot-topic pathways in microspheroids of the A549 cell line (**A**) and Colo699 cell line (**B**) Red indicates in total an up-regulation of involved genes, whereas blue depicts an overall down regulation within the specific pathway. NES describes the normalized enrichment scores and FDR the false discovery rate.

Furthermore, specific gene signature enrichment analyses were run to investigate and depict up- or down-regulation due to culture technique of clinically and scientifically hot topic pathways via xtools.gsea. For the A549 significantly up-regulated gene signatures included immunological/inflammatory pathways and endothelial proliferation (Figure 6A). Most of these pathways were also found to be up-regulated in Colo699, even though they did not reach significance. On the other side, unexpectedly several pathways, such as hypoxia, tumor invasion, p53-signaling, EMT and TGF-beta, as well as angiogenesis (Figure 6A) were down regulated in A549 cells, with concordant findings in Colo699 for cell cycle, p53 signaling and hypoxia (Figure 6B).

### *In-vivo* data comparison

Based on the hypothesis of this work that 3D cell cultivation not only alters the gene expression patterns, but also mimics the *in-vivo* situation more accurate, we identified patient derived data sets in The Cancer Genome Atlas (TCGA) of tumor versus normal tissue samples and metastatic samples versus primary tumors.

**Figure 7:**
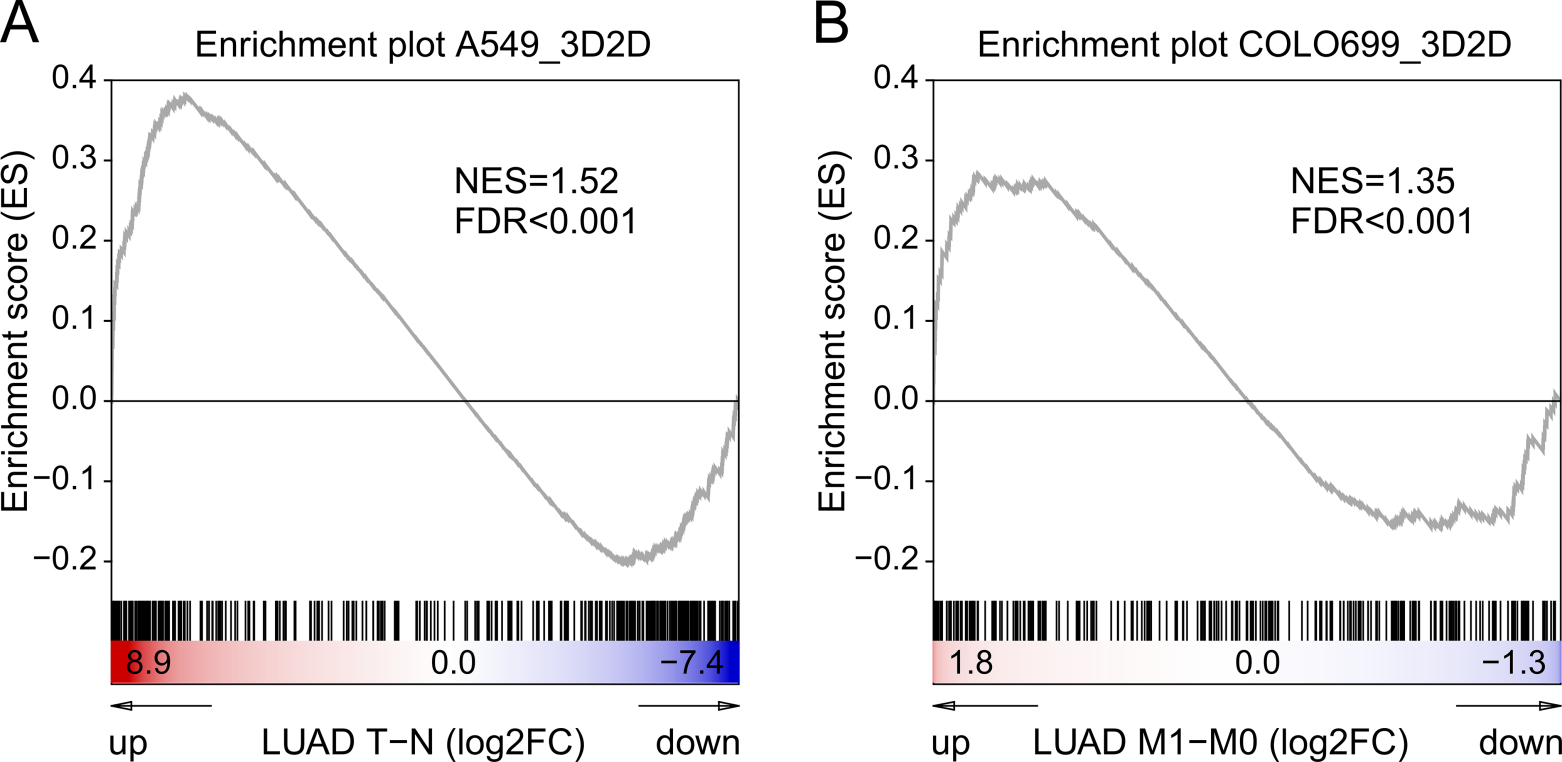
Gene set enrichment analyses 3D versus *in-vivo* tumor samples The enrichment plots depict gene set enrichment analyses of 3D vs 2D up-regulated genes (at least 2FC) in comparison to published NSCLC patient derived data from The Cancer Genome Atlas. For the A549 the regulated gene profiles of the 3D system were tested for their enrichment in tumour tissues compared to normal tissues (LUAD data set; *n* = 58) (**A**), whereas in the analyses of Colo699 the 3D profile was compared to changes between metastases and primary tumours (**B**).

We ran a gene set enrichment analysis for the regulated genes (> 2FC expression change) of our 3D to 2D comparison in these patient-derived data sets. For the A549 cell line the 3D altered gene profile was compared to data from tumor versus normal tissues (*n* = 58) (Figure 7A). For the Colo699, due to their metastatic origin, we compared their profile to metastatic samples versus primary tumors (M0: *n* = 347, M1: *n* = 25) (Figure 7B). In both cases we were able to identify a statistical significant enrichment of our 3D-gene profiles in the *in-vivo* setting.

## DISCUSSION

The frequent failures of targeted drugs in late drug development lead to the establishment of several 3-dimensional cancer models for more *in-vivo* like conditions. Several recent publications showed a difference in drug sensitivity for 3D models [5, 12, 17, 18], suggesting of course that alteration in protein and RNA/gene expressions lead to drug resistance.

Our work now confirms changes of RNA expression profiles in a hanging drop 3D culture system.

The hanging drop model was chosen, as the system enables long time cultivation, repeated drug application and medium exchange in comparison to other 3D culture methods [5, 8]. In addition, no external influencing factors, such as growth factors or artificial skeleton/structures, need to be added in our system [19].

Our analyses proved a high reproducibility in three independent experiments providing a high validity for our results.

For this work two adenocarcinoma cell lines, the A549, as primary-derived tumor cell line, and the Colo699 with metastatic origin from pleural effusion were chosen. Thus, the primarily observed small overlap of changes in gene expression (see Supplementary Figure 2) and their clear separation within the component analyses (Figure 2) between these two tumor cell lines might reflect their difference in origin and over all tumor heterogeneity. This further supports the need for innovative drug testing systems reflecting individual tumors.

Concerning the altered genes illustrated in Tables 2 and 3 most have been described to play a role in cancer progression, invasiveness and metastases. For example the CEA-family with CEACAM 5 & 6 are well established tumor markers for CRC. Both were highly up-regulated in the A549 cell line during 3D cultivation and have been associated with cancer progression and metastases [20, 21]. Nevertheless, the pathway analyses revealed a down regulation for the tumor invasiveness in A549 cells. CEACAM 1 was also found to be up-regulated in both cell lines during 3D cultivation and described to be responsible for the angiogenic potential of lung cancers [22]. Others, such as the BPIFA1 have been associated with lung disease, as well as nasopharyngeal carcinomas [23]. F5, as part of the coagulation cascade, might be involved in the hyper-coagulation in solid tumor patients and their prognosis [24].

Besides the small number of overlapping gene alterations between the cell lines, the thereby affected pathways showed a great overlap, suggesting the underlying molecular mechanisms to be more comparable for both cell lines.

Most of these regulated pathways are known key cancer pathways, underlining the importance of the 3D cultivation technique and supporting the results stated by others [25].

Besides growth and cell division affecting pathways, also epigenetic alterations including DNA methylation are affected. These changes are known to play a role in transforming processes during carcinogenesis and some of them are also described in lung cancer [26–29]. Several of the other altered pathways, such as DNA damage repair [30], rhoGTPase [31], Wnt/ß-catenin with PKN1 [32, 33] and notch with SIRT1 [34–36] signaling pathways, are involved in cancerogenesis in general and described in lung cancers. They were associated with outcomes, invasiveness/aggressiveness and drug resistance. In particular, SIRT1 has been associated with poor prognosis in lung cancer patients [34]. The Wnt-signalling pathway has been associated with rhoGTPase regulation [37] and notch signaling in lung cancer [38]. This highlights another indication for the usage of our 3D cell model – to investigate the cross talks of pathways – for example between wnt, rhoGTPase and notch signaling.

In another analysis we focused on specific pathways with actual clinical relevance [39–41]. Thereby, the immunological pathways, endothelial proliferation and angiogenesis, hypoxia, invasiveness, EMT and TGF-beta, but also cell cycle and p53 signaling were found to be affected by 3D cultivation in our gene enrichment analyses. During the last years, especially the immune system and its alterations have become a hot topic for translational research with several new agents targeting tumor-immune system interactions [40, 41]. Thus, the up-regulation of the innate and adaptive immune response in A549 cells cultured in 3D is of major interest and suggests the use of the 3D culture system to address immunological issues and drug testing, as also stated for various applications by others [42]. Some of these described cancer pathways were found unexpectedly down-regulated in our 3D cultures. This includes EMT, hypoxia, angiogenesis and the p53 pathway. These pathways are associated with tumor aggressiveness [43], invasion [44, 45] and drug resistance [46, 47]. Their down-regulations warrant mechanistic investigations with a more detailed examination of the role and impact of specific genes within these pathways. These findings also need to be considered in future works and suggest bigger spheroids to induce hypoxia or co-culture models with fibroblasts and/or endothelial cells. By these co-cultures a denser spheroid structure and cell-cell interactions can be achieved. These play an important role in EMT and hypoxia [48, 49].

In addition, changes in proliferation due to differences between outer and inner zones might contribute to gene regulation. Nevertheless, the expression of ki67 was low in A549 monocultures and evenly distributed in the Colo699 microtissues, as shown before. Hence, the confounding effect of various proliferation zones seems limited in these cultures and might as well occur in *in-vivo* samples.

Several other groups have investigated other 3D cell culture models [13, 50, 51]: In contrast to our findings, analyses of A549 cells cultured in matrigel showed no differences in DNA repair mechanisms [14] and a down regulation of the immune response and complement system in GO analyses. Nevertheless, other findings of this work were concordant, such as the down-regulation of NFkappa B [15].

This underlines the influence of the used culture technique and culture duration, as these works used different time points for their analyses (4 days or 15 days, respectively). The influence of cultivation duration was also indicated in our comparison of 5 versus 10 days, even though the differences in affected pathways analyses were limited. Furthermore, matrigel is an artificial ECM enriched with growth factors and thereby induces signaling cascades that are not activated in the hanging drop technology.

These issues should be regarded in future microtissue applications and investigations.

In summary, our experiments highlight the relevance of a 3D cultivation technique, but also warrant further investigations based on the high variability of the gene expressions between these two cell lines, as well as the partially unexpected down regulations, for example within hypoxic and EMT pathways. These findings in combination with our *in-vivo* comparisons support the importance of such models in mechanistic studies and early drug development in general to close the gap between *in-vitro* and *in-vivo* conditions, but underline the need to individualize and consider potential limitations of each model.

## MATERIALS AND METHODS

### Cell culture

The human non-small cell lung cancer cell line Colo699 (DSMZ, ACC196) and A549 (DSMZ, ACC-107), which were authenticated using STR-profiling (see Supplementary Table 1) were used.

Cells were either cultivated as monolayers in standard 12-well plates (PAA laboratories GmbH, Germany) or in the later described 3D system. DMEM low glucose (Lonza Group AG-REG, Swiss) supplemented with 10% fetal calf serum (FCS) (Sigma-Aldrich, Germany) and 100 U/ml penicillin, 100 mg/ml streptomycin solution, 2 mM L-Glutamine (PAA laboratories GmbH, Germany) was used as medium for 2D and 3D cultivation.

For RNA isolation 75,000 cells were cultivated in 12 well plates for 5 days, or 96 spheroids were pooled after 5 or 10 days, respectively. Their morphological appearance (spheroid formation) was assessed via light microscopy prior to pooling to exclude dead or unformed spheroids (see also Figure 1A).

### Microtissue culture

Spheroids were grown in the Gravity-PLUSTM microtissue culture system (InSphero AG, Zurich, Switzerland), as described earlier [8]. In short, 2,500 tumor cells from subconfluently 2D-grown cells (A549, Colo699) were seeded in the hanging drop plates. Therefore, 2D grown cells were detached with accutase (PAA laboratories GmbH, Germany) for five minutes at 37°C. After dilution with medium cells were counted and seeded in 40 μl drops at a density of 2,500 cells/drop.

Both, cells and microtissues were cultivated at 37°C in a humidified 5% CO2-containing atmosphere. Morphological appearances of the A549 and Colo699 3D microtissues were controlled via an Olympus IX70 (Japan) inverted light microscope.

### Morphological and viability assessment

The LIVE/DEAD^®^ Viability/Cytotoxicity Kit for mammalian cells“ (Thermo Fisher Scientific, USA) was performed according to manufacturer‘s instructions with some adoptions for our 3D cell culture system to stain for live and dead cells in our spheroids in the hanging drop on day 5, 7, 10 and 14. Stock solutions from calcein AM (CaAM, 4 mM, dye for live cells) and ethidium homodimer-1 (EthD-1, 2 mM, dye for dead cells) were diluted together with PBS (Lonza, Germany) to concentrations of 15 μM (CaAM) and 20 μM (EthD-1). In the next step, 20 μL of media were removed from the hanging drop and replaced with 20 μL of the staining solution. The final concentration for CaAM was 7.5 μM and for EthD-1 10 μM. After an incubation time of 1 hour at 37°C, stained spheroids were analyzed using the DMi8 inverted microscope (Leica, Germany) with the LAS X 1.1.0 software (Leica, Germany) (magnification: 100x). Single stainings of spheroids were used to establish concentration of dyes and settings for the microscope.

### RNA isolation and quality assessment

To demonstrate RNA expression differences, cells cultured in the 12-well 2D setting for 5 days, as well as those cultured in the 3D hanging drop system for five and ten days, were harvested and washed with PBS. Total RNA was isolated from pooled spheroids (96 spheroids/RNA isolation) and cells (75,000/well) using the Trizol protocol (TriReagent, Sigma Aldrich T9424). Quantity and integrity of RNA were assessed by optical density measurements using the nanodrop system (ThermoScientific Nano Drop 2000) and the 2100 Bioanalyzer (Agilent Technologies, Palo Alto, CA) according to the manufacturer's protocols. RNA samples with a 260/280 ratio > 1.85, a 230/260 ratio > 2.0 and RIN-values between 9.7 to 10 were used in subsequent analyses.

### Affymetrix chip analyses

The whole-genome gene expression data set consisting of in total 15 microarrays (3 replicates per sample group) was generated at the Expression Profiling Unit of the Medical University of Innsbruck. In brief, 250 ng of high quality RNA per sample were processed using standard protocols employing the Affymetrix GeneChip WT Expression kit and Terminal labelling kit. The resulting biotinylated targets were hybridized to Affymetrix HuGene 1.0 ST microarrays, which, after staining in an Affymetrix fluidic station, were scanned in an Affymetrix scanner 3000.

All further analyses were performed in R (version 3.2.2) using packages from the Bioconductor project (version 3.2) [52]. Pre-processing of the raw microarray data was performed as described before [53]. In brief, pre-processing was performed using the general gcrma package [54] and employing a custom CDF that defines a probe set for each transcript of all genes defined in Ensembl version 75. Raw and pre-processed data have been deposited in the Gene Expression Omnibus (GEO, accession number: GSE78210).

These data are accessible by using the following link: http://www.ncbi.nlm.nih.gov/geo/query/acc.cgi?token=ovcpsumanzivlsf&acc=GSE782369 10.

Differential expression analysis was performed using the limma package [55]. The resulting *p*-values were adjusted for multiple hypothesis testing according to the Benjamini and Hochberg method [56]. Genes with an average M-value larger than 1 (more than 2-fold regulated) and an adjusted *p*-value smaller than 0.01 (1% false discovery rate) were considered to differ significantly in their expression.

### Quantitative RT-PCR analysis to validate microarray results

To validate results of the microarray experiments, four significantly regulated genes were tested in additional qPCR analyses. Total mRNA was isolated, as described earlier from pooled microspheroids of three independent experiments. Genomic DNA in the RNA samples was digested with DNAse I (New England Biolabs). The cDNA was amplified from 1 μg total RNA using the SuperScript II Reverse Transcriptase Kit (Invitrogen Life Technologies). Quantitative real time polymerase chain reaction (RT-PCR) was performed using a SensiMix SYBR No-ROX Kit (Bioline), a Rotor-Gene 6000 detection system (Corbett Research) and sets of gene-specific primers (Table 1). The target-specific primers were generated using the NCBI Primer-BLAST. Ct values were determined using the Rotor-Gene 6000 Series Software 1.7. Expression ratios were calculated using the ΔΔCt-method. As reference gene, 18S-rRNA was chosen.

### Pathway and Gene Ontology enrichment analysis

We employed the hypergeometric testing framework of Bioconductor's Category and GOstats packages [57] to identify pathways or biological processes with a significant enrichment of differentially expressed genes. All genes detectable on the microarray were used as background gene set as suggested by Rhee et al. [58] and the conditional test [57] was used for the gene ontology analysis to enrich for more specific terms/biological processes. The resulting *p*-values were adjusted using the Bonferroni method and all pathways or biological processes with a *p*-value smaller than 0.05 were considered to be significantly enriched. Reactome pathway definitions and Gene Ontology annotations used Bioconductor's reactome.db (version 1.54.1) and GO.db (version 3.2.2).

### Gene set enrichment analyses (GSEA)

We pre-ranked genes for the A549 and the Colo699 cell line based on differential expression between 3D and 2D cultures by log2 fold changes. To identify which gene sets of the major cancer pathways are enriched in the 3D vs 2D cell culture, we applied the GSEA software [59] (Broad Java web start implementation) on these pre-ranked gene lists using designated gene sets (hallmark collection from the molecular signature database (MSigDB) and other common cancer signatures/signaling pathways). Standard settings with 1000 runs of gene permutations were employed. To compare the different gene expression between the 3D and 2D culture to *in vivo* profiles we downloaded RNAseq data (level 3 RNAseq V2) from LUAD samples of The Cancer Genome Atlas (TCGA) via Firehose/Firebrowse. Differential gene expression for paired tumor and normal samples (*n* = 58) and for metastatic samples (M1; *n* = 25) versus none metastatic samples (M0; *n* = 347) were determined using DESeq2 [60]. We tested the up-regulated genes (> 2FC) from A549 3D vs. 2D culture, using the same procedure described above, for their enrichment in this pre-ranked gene list from tumor versus paired normal tissue. As Colo699 originate from metastatic cancer the significantly up-regulated genes (> 2FC) were compared to the pre-ranked gene list of differentially expressed ones between metastatic vs. none metastatic samples. Customized R scripts were used for all further visualizations.

## CONCLUSIONS

Anticancer drug development is moving towards individualized biomarker-driven therapy, which urges the need for models reflecting *in-vivo* conditions.

Microtissues with their tumor microenvironment are known to partially meet these requirements [17] along with extended cultivation and incubation times.

With this work, we proved data concerning relevant alterations in gene expression between 2D and 3D cultivation methods. The found differences affect most relevant cancer-associated and resistance-mediating pathways, even though some warrant future mechanistic investigations. The genes, which were altered by our 3D culture techniques, were enriched in patient-derived data of The Cancer Genome Atlas, underlining the potential of this model.

## SUPPLEMENTARY MATERIALS FIGURES AND TABLES



## References

[1] Suggitt M, Bibby MC (2005). 50 years of preclinical anticancer drug screening: empirical to target-driven approaches. Clin Cancer Res.

[2] Hait WN (2010). Anticancer drug development: the grand challenges. Nat Rev Drug Discov.

[3] Subramanian J, Regenbogen T, Nagaraj G, Lane A, Devarakonda S, Zhou G, Govindan R (2013). Review of ongoing clinical trials in non-small-cell lung cancer: a status report for 2012 from the ClinicalTrials.gov Web site. J Thorac Oncol.

[4] Garcia VM, Cassier PA, de Bono J (2011). Parallel anticancer drug development and molecular stratification to qualify predictive biomarkers: dealing with obstacles hindering progress. Cancer Discov.

[5] Amann A, Gamerith G, Huber JM, Zwierzina M, Hilbe W, Zwierzina H (2015). Predicting drug sensitivity by 3D cell culture models. Memo.

[6] Xu X, Farach-Carson MC, Jia X (2014). Three-dimensional *in vitro* tumor models for cancer research and drug evaluation. Biotechnol Adv.

[7] Phung YT, Barbone D, Broaddus VC, Ho M (2011). Rapid generation of *in vitro* multicellular spheroids for the study of monoclonal antibody therapy. J Cancer.

[8] Amann A, Zwierzina M, Gamerith G, Bitsche M, Huber JM, Vogel GF, Blumer M, Koeck S, Pechriggl EJ, Kelm JM, Hilbe W, Zwierzina H (2014). Development of an innovative 3D cell culture system to study tumour—stroma interactions in non-small cell lung cancer cells. PLoS One.

[9] Achilli TM, Meyer J, Morgan JR (2012). Advances in the formation, use and understanding of multi-cellular spheroids. Expert Opin Biol Ther.

[10] Pampaloni F, Reynaud EG, Stelzer EH (2007). The third dimension bridges the gap between cell culture and live tissue. Nat Rev Mol Cell Biol.

[11] Asthana A, Kisaalita WS (2012). Microtissue size and hypoxia in HTS with 3D cultures. Drug Discov Today.

[12] Lovitt CJ, Shelper TB, Avery VM (2014). Advanced cell culture techniques for cancer drug discovery. Biology (Basel).

[13] Däster S, Amatruda N, Calabrese D, Ivanek R, Turrini E, Droeser RA, Zajac P, Fimognari C, Spagnoli GC, Iezzi G, Mele V, Muraro MG (2017). Induction of hypoxia and necrosis in multicellular tumor spheroids is associated with resistance to chemotherapy treatment. Oncotarget.

[14] Zschenker O, Streichert T, Hehlgans S, Cordes N (2012). Genome-wide gene expression analysis in cancer cells reveals 3D growth to affect ECM and processes associated with cell adhesion but not DNA repair. PLoS One.

[15] Mishra DK, Creighton CJ, Zhang Y, Gibbons DL, Kurie JM, Kim MP (2014). Gene expression profile of A549 cells from tissue of 4D model predicts poor prognosis in lung cancer patients. Int J Cancer.

[16] Rimann M, Laternser S, Gvozdenovic A, Muff R, Fuchs B, Kelm JM, Graf-Hausner U (2014). An *in vitro* osteosarcoma 3D microtissue model for drug development. J Biotechnol.

[17] Ma HL, Jiang Q, Han S, Wu Y, Cui Tomshine J, Wang D, Gan Y, Zou G, Liang XJ (2012). Multicellular tumor spheroids as an *in vivo*-like tumor model for three-dimensional imaging of chemotherapeutic and nano material cellular penetration. Mol Imaging.

[18] Huber JM, Amann A, Koeck S, Lorenz E, Kelm JM, Obexer P, Zwierzina H, Gamerith G (2016). Evaluation of assays for drug efficacy in a three-dimensional model of the lung. J Cancer Res Clin Oncol.

[19] Thoma CR, Zimmermann M, Agarkova I, Kelm JM, Krek W (2014). 3D cell culture systems modeling tumor growth determinants in cancer target discovery. Adv Drug Deliv Rev.

[20] Beauchemin N, Arabzadeh A (2013). Carcinoembryonic antigen-related cell adhesion molecules (CEACAMs) in cancer progression and metastasis. Cancer Metastasis Rev.

[21] Benlloch S, Galbis-Caravajal JM, Alenda C, Peiró FM, Sanchez-Ronco M, Rodríguez-Paniagua JM, Baschwitz B, Rojas E, Massutí B (2009). Expression of molecular markers in mediastinal nodes from resected stage I non-small-cell lung cancer (NSCLC): prognostic impact and potential role as markers of occult micrometastases. Ann Oncol.

[22] Dango S, Sienel W, Schreiber M, Stremmel C, Kirschbaum A, Pantel K, Passlick B (2008). Elevated expression of carcinoembryonic antigen-related cell adhesion molecule 1 (CEACAM-1) is associated with increased angiogenic potential in non-small-cell lung cancer. Lung Cancer.

[23] Zhang W, Zeng Z, Wei F, Chen P, Schmitt DC, Fan S, Guo X, Liang F, Shi L, Liu Z, Zhang Z, Xiang B, Zhou M (2014). SPLUNC1 is associated with nasopharyngeal carcinoma prognosis and plays an important role in all-trans-retinoic acid-induced growth inhibition and differentiation in nasopharyngeal cancer cells. FEBS J.

[24] Tas F, Kilic L, Serilmez M, Keskin S, Sen F, Duranyildiz D (2013). Clinical and prognostic significance of coagulation assays in lung cancer. Respir Med.

[25] Pampaloni F, Reynaud EG, Stelzer EH (2007). The third dimension bridges the gap between cell culture and live tissue. Nat Rev Mol Cell Biol.

[26] Heyn H, Vidal E, Ferreira HJ, Vizoso M, Sayols S, Gomez A, Moran S, Boque-Sastre R, Guil S, Martinez-Cardus A, Lin CY, Royo R, Sanchez-Mut JV (2016). Epigenomic analysis detects aberrant super-enhancer DNA methylation in human cancer. Genome Biol.

[27] Lin SH, Wang J, Saintigny P, Wu CC, Giri U, Zhang J, Menju T, Diao L, Byers L, Weinstein JN, Coombes KR, Girard L, Komaki R (2014). Genes suppressed by DNA methylation in non-small cell lung cancer reveal the epigenetics of epithelial-mesenchymal transition. BMC Genomics.

[28] Balgkouranidou I, Liloglou T, Lianidou ES (2013). Lung cancer epigenetics: emerging biomarkers. Biomarkers Med.

[29] Mehta A, Dobersch S, Romero-Olmedo AJ, Barreto G (2015). Epigenetics in lung cancer diagnosis and therapy. Cancer Metastasis Rev.

[30] Bonanno L, Favaretto A, Rosell R (2014). Platinum drugs and DNA repair mechanisms in lung cancer. Anticancer Res.

[31] Vega FM, Ridley AJ (2008). Rho GTPases in cancer cell biology. FEBS Lett.

[32] James RG, Bosch KA, Kulikauskas RM, Yang PT, Robin NC, Toroni RA, Biechele TL, Berndt JD, von Haller PD, Eng JK, Wolf-Yadlin A, Chien AJ, Moon RT (2013). Protein kinase PKN1 represses Wnt/β-catenin signaling in human melanoma cells. J Biol Chem.

[33] Jilg CA, Ketscher A, Metzger E, Hummel B, Willmann D, Rüsseler V, Drendel V, Imhof A, Jung M, Franz H, Hölz S, Krönig M, Müller JM, Schüle R (2014). PRK1/PKN1 controls migration and metastasis of androgen-independent prostate cancer cells. Oncotarget.

[34] Grbesa I, Pajares MJ, Martínez-Terroba E, Agorreta J, Mikecin AM, Larráyoz M, Idoate MA, Gall-Troselj K, Pio R, Montuenga LM (2015). Expression of sirtuin 1 and 2 is associated with poor prognosis in non-small cell lung cancer patients. PLoS One.

[35] Lin Z, Fang D (2013). The Roles of SIRT1 in Cancer. Genes Cancer.

[36] Xie M, Liu M, He CS (2012). SIRT1 regulates endothelial Notch signaling in lung cancer. PLoS One.

[37] Schlessinger K, Hall A, Tolwinski N (2009). Wnt signaling pathways meet Rho GTPases. Genes Dev.

[38] Mazieres J, He B, You L, Xu Z, Jablons DM (2005). Wnt signaling in lung cancer. Cancer Lett.

[39] Byers LA, Diao L, Wang J, Saintigny P, Girard L, Peyton M, Shen L, Fan Y, Giri U, Tumula PK, Nilsson MB, Gudikote J, Tran H (2012). An epithelial-mesenchymal transition gene signature predicts resistance to EGFR and PI3K inhibitors and identifies Axl as a therapeutic target for overcoming EGFR inhibitor resistance. Clin Cancer Res.

[40] Farkona S, Diamandis EP, Blasutig IM (2016). Cancer immunotherapy: the beginning of the end of cancer?. BMC Med.

[41] Reck M, Rodríguez-Abreu D, Robinson AG, Hui R, Csőszi T, Fülöp A, Gottfried M, Peled N, Tafreshi A, Cuffe S, O’Brien M, Rao S, Hotta K, KEYNOTE-024 Investigators (2016). Pembrolizumab versus Chemotherapy for PD-L1-Positive Non-Small-Cell Lung Cancer. N Engl J Med.

[42] Koeck S, Zwierzina M, Lorenz E, Gamerith G, Zwierzina H, Amann A (2015). Infiltration of immune cells into cancer cell/stroma cell 3D microtissues. J Immunother Cancer.

[43] da Motta LL, De Bastiani MA, Stapenhorst F, Klamt F (2015). Oxidative stress associates with aggressiveness in lung large-cell carcinoma. Tumour Biol.

[44] Mani SA, Guo W, Liao MJ, Eaton EN, Ayyanan A, Zhou AY, Brooks M, Reinhard F, Zhang CC, Shipitsin M, Campbell LL, Polyak K, Brisken C (2008). The epithelial-mesenchymal transition generates cells with properties of stem cells. Cell.

[45] Yang J, Weinberg RA (2008). Epithelial-mesenchymal transition: at the crossroads of development and tumor metastasis. Dev Cell.

[46] Thomson S, Buck E, Petti F, Griffin G, Brown E, Ramnarine N, Iwata KK, Gibson N, Haley JD (2005). Epithelial to mesenchymal transition is a determinant of sensitivity of non-small-cell lung carcinoma cell lines and xenografts to epidermal growth factor receptor inhibition. Cancer Res.

[47] Pircher A, Jöhrer K, Kocher F, Steiner N, Graziadei I, Heidegger I, Pichler R, Leonhartsberger N, Kremser C, Kern J, Untergasser G, Gunsilius E, Hilbe W (2016). Biomarkers of evasive resistance predict disease progression in cancer patients treated with antiangiogenic therapies. Oncotarget.

[48] Mendes F, Sales T, Domingues C, Schugk S, Abrantes AM, Gonçalves AC, Teixo R, Silva R, Casalta-Lopes J, Rocha C, Laranjo M, Simões PC, Ribeiro AB (2015). Effects of X-radiation on lung cancer cells: the interplay between oxidative stress and P53 levels. Med Oncol.

[49] Amann A, Zwierzina M, Koeck S, Gamerith G, Pechriggl E, Huber JM, Lorenz E, Kelm JM, Hilbe W, Zwierzina H, Kern J (2017). Development of a 3D angiogenesis model to study tumour - endothelial cell interactions and the effects of anti-angiogenic drugs. Sci Rep.

[50] Breslin S, O’Driscoll L (2016). The relevance of using 3D cell cultures, in addition to 2D monolayer cultures, when evaluating breast cancer drug sensitivity and resistance. Oncotarget.

[51] Jakubikova J, Cholujova D, Hideshima T, Gronesova P, Soltysova A, Harada T, Joo J, Kong SY, Szalat RE, Richardson PG, Munshi NC, Dorfman DM, Anderson KC (2016). A novel 3D mesenchymal stem cell model of the multiple myeloma bone marrow niche: biologic and clinical applications. Oncotarget.

[52] Huber W, Carey VJ, Gentleman R, Anders S, Carlson M, Carvalho BS, Bravo HC, Davis S, Gatto L, Girke T, Gottardo R, Hahne F, Hansen KD (2015). Orchestrating high-throughput genomic analysis with Bioconductor. Nat Methods.

[53] Bindreither D, Ecker S, Gschirr B, Kofler A, Kofler R, Rainer J (2014). The synthetic glucocorticoids prednisolone and dexamethasone regulate the same genes in acute lymphoblastic leukemia cells. BMC Genomics.

[54] Rainer J, Lelong J, Bindreither D, Mantinger C, Ploner C, Geley S, Kofler R (2012). Research resource: transcriptional response to glucocorticoids in childhood acute lymphoblastic leukemia. Mol Endocrinol.

[55] Smyth GK (2004). Linear models and empirical bayes methods for assessing differential expression in microarray experiments. Stat Appl Genet Mol Biol.

[56] Benjamini Y, Hochberg Y (1995). Controlling the False Discovery Rate: A Practical and Powerful Approach to Multiple Testing. J R Stat Soc Ser B-. Stat Methodol.

[57] Falcon S, Gentleman R (2007). Using GOstats to test gene lists for GO term association. Bioinformatics.

[58] Rhee SY, Wood V, Dolinski K, Draghici S (2008). Use and misuse of the gene ontology annotations. Nat Rev Genet.

[59] Subramanian A, Tamayo P, Mootha VK, Mukherjee S, Ebert BL, Gillette MA, Paulovich A, Pomeroy SL, Golub TR, Lander ES, Mesirov JP (2005). Gene set enrichment analysis: a knowledge-based approach for interpreting genome-wide expression profiles. Proc Natl Acad Sci USA.

[60] Love MI, Huber W, Anders S (2014). Moderated estimation of fold change and dispersion for RNA-seq data with DESeq2. Genome Biol.

