# Supplementary material for: 3D-cultivation of NSCLC cell lines induce gene expression alterations of key cancer-associated pathways and mimic in-vivo conditions
Source: Oncotarget. 2017 Nov 6;8(68):112647–61. doi: 10.18632/oncotarget.22636 (PMC5762538; doi:10.18632/oncotarget.22636)
Supplement: Supplementary file 1 [file oncotarget-08-112647-s001.pdf]

3D-cultivation of NSCLC cell lines induce gene expression alterations of key cancer-associated pathways and mimic in-vivo conditions

SUPPLEMENTARY MATERIALS

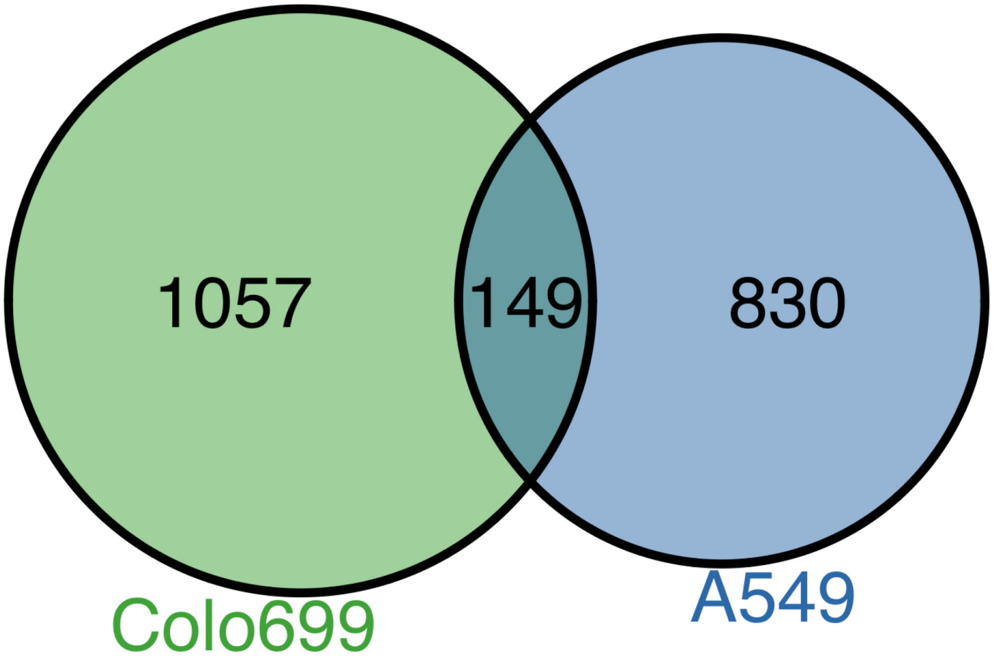

Supplementary Figure 1: Venn diagram comparing the numbers of significantly differentially expressed genes between 3D and 2D cell culture condition in A549 and Colo699 cells.

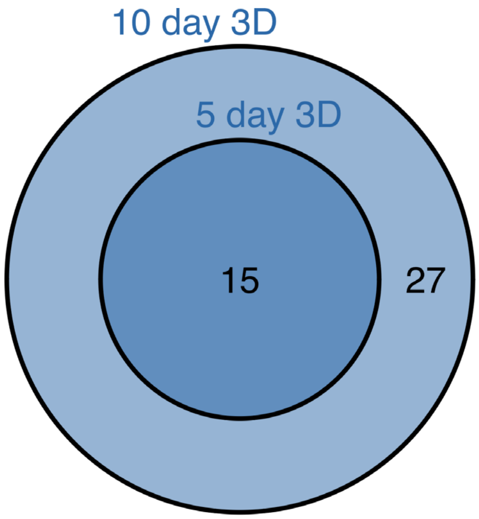

(a) Enriched reactome pathways.

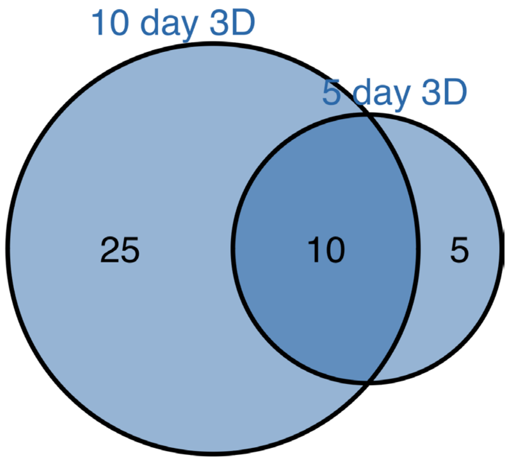

(b) Enriched GO biological processes.

Supplementary Figure 2: Venn diagram comparing the numbers enriched pathways or GO terms for the comparison of 10 day 3D vs 5 day 2D (10 day 3D) cultures or 5 day 3D vs 5 day 2D (5 day 3D) cultures of A549 cells.

Supplementary Table 1: STR Profiles of the two cell lines A549 and Colo-699

**Marker**<br>**Cell line**	**D5S818**	**D13S317**	**D7S820**	**D16S539**	**VWA**	**TH01**	**AM**	**TPOX**	**CSF1PO**
Used cell line	11, 11	11, 11	8, 11	11, 12	13, 14	8, 9.3	×, ×	8, 11	10, 12
A549	11, 11	11, 11	8, 11	11, 12	13, 14	8, 9.3	×, ×	8, 11	10, 12
Used cell line	11, 12	11, 11	11, 13	12, 12	17, 17	9, 9.3	×, ×	8, 8	8, 8
COLO-699	11, 12	11, 11	11, 13	12, 12	17, 17	9, 9.3	×, ×	8, 8	8, 8

Supplementary Table 2: Pathways with significant overrepresentation of genes differentially expressed between 3D and 2D cultures of A549 cells

Pathway name	padj	Count	Size
DNA methylation	0.000	24	30
Activated PKN1 stimulates transcription of AR (androgen receptor) regulated genes KLK2 and KLK3	0.000	24	33
SIRT1 negatively regulates rRNA Expression	0.000	24	34
Meiotic recombination	0.000	28	50
Amyloids	0.000	28	50
PRC2 methylates histones and DNA	0.000	24	39
RHO GTPases activate PKNs	0.000	26	56
Meiosis	0.000	30	80
Senescence-Associated Secretory Phenotype (SASP)	0.000	27	68
Condensation of Prophase Chromosomes	0.000	17	26
RHO GTPase Effectors	0.000	46	227
Packaging Of Telomere Ends	0.000	14	20
NoRC negatively regulates rRNA expression	0.000	24	69
Transcriptional regulation by small RNAs	0.000	24	69
Negative epigenetic regulation of rRNA expression	0.000	24	72
Signaling by Rho GTPases	0.000	55	340
Oxidative Stress Induced Senescence	0.000	25	84
Regulatory RNA pathways	0.000	26	96
DNA Damage/Telomere Stress Induced Senescence	0.000	16	36
RMTs methylate histone arginines	0.000	15	32
Deposition of new CENPA-containing nucleosomes at the centromere	0.000	17	43
Nucleosome assembly	0.000	17	43
Epigenetic regulation of gene expression	0.000	24	88
HDMs demethylate histones	0.000	15	34
Meiotic synapsis	0.000	17	47
HATs acetylate histones	0.000	27	118
Cellular Senescence	0.000	29	137
formation of the beta-catenin:TCF transactivating complex	0.000	16	44
Cell Cycle	0.000	60	482
PKMTs methylate histone lysines	0.000	15	49
HDACs deacetylate histones	0.000	14	44
Telomere Maintenance	0.000	14	45
Mitotic Prophase	0.000	18	75
Chromosome Maintenance	0.001	17	74
Hemostasis	0.002	51	436
Response to elevated platelet cytosolic Ca2+	0.004	17	84
Chromatin modifying enzymes	0.006	30	214
Chromatin organization	0.006	30	214
Platelet degranulation	0.007	16	79
TCF dependent signaling in response to WNT	0.011	25	168
Cell Cycle, Mitotic	0.013	45	398
M Phase	0.046	29	228

Columns p adj, Count and Size contain the Bonferroni adjusted *p*-values, the number of differentially expressed genes in the pathway and the total number of genes in the pathway, respectively.

Supplementary Table 3: Pathways with significant overrepresentation of genes differentially expressed between 3D and 2D cultures of Colo699 cells

Pathway name	padj	Count	Size
Cell Cycle	0.000	111	482
Cell Cycle, Mitotic	0.000	90	398
SIRT1 negatively regulates rRNA Expression	0.000	26	34
Meiotic recombination	0.000	31	50
DNA methylation	0.000	24	30
Activated PKN1 stimulates transcription of AR (androgen receptor) regulated genes KLK2 and KLK3	0.000	24	33
PRC2 methylates histones and DNA	0.000	25	39
Condensation of Prophase Chromosomes	0.000	20	26
Deposition of new CENPA-containing nucleosomes at the centromere	0.000	25	43
Nucleosome assembly	0.000	25	43
Meiosis	0.000	33	80
RHO GTPase Effectors	0.000	57	227
Signaling by Rho GTPases	0.000	72	340
Amyloids	0.000	26	50
Chromosome Maintenance	0.000	31	74
Senescence-Associated Secretory Phenotype (SASP)	0.000	29	68
RHO GTPases activate PKNs	0.000	26	56
M Phase	0.000	52	228
HATs acetylate histones	0.000	35	118
Negative epigenetic regulation of rRNA expression	0.000	27	72
NoRC negatively regulates rRNA expression	0.000	26	69
Oxidative Stress Induced Senescence	0.000	28	84
Packaging Of Telomere Ends	0.000	14	20
Mitotic Prometaphase	0.000	30	99
Telomere Maintenance	0.000	20	45
Epigenetic regulation of gene expression	0.000	28	88
Resolution of Sister Chromatid Cohesion	0.000	28	91
Transcriptional regulation by small RNAs	0.000	24	69
DNA Damage/Telomere Stress Induced Senescence	0.000	16	36
RMTs methylate histone arginines	0.000	15	32
RHO GTPases Activate Formins	0.000	27	101
HDMs demethylate histones	0.000	15	34
Mitotic Prophase	0.000	22	75
Cellular Senescence	0.000	31	137
Regulatory RNA pathways	0.000	25	96
Meiotic synapsis	0.000	17	47
Chromatin modifying enzymes	0.000	40	214
Chromatin organization	0.000	40	214
PKMTs methylate histone lysines	0.000	17	49
Polo-like kinase mediated events	0.000	9	16
Cholesterol biosynthesis	0.000	10	21
G2/M Checkpoints	0.001	15	50
formation of the beta-catenin:TCF transactivating complex	0.001	14	44
HDACs deacetylate histones	0.001	14	44
Separation of Sister Chromatids	0.001	28	148
Factors involved in megakaryocyte development and platelet production	0.002	23	112
Cyclin A/B1 associated events during G2/M transition	0.002	8	16
Mitotic Anaphase	0.002	28	156
Activation of ATR in response to replication stress	0.002	12	37
Mitotic Metaphase and Anaphase	0.003	28	157
E2F mediated regulation of DNA replication	0.004	10	27
Phosphorylation of proteins involved in G1/S transition by active Cyclin E:Cdk2 complexes	0.009	4	4
Activation of the pre-replicative complex	0.010	10	30
Mitotic G1-G1/S phases	0.015	22	120
Cellular responses to stress	0.015	36	248
G2/M Transition	0.028	20	108
G1/S-Specific Transcription	0.028	6	12
Kinesins	0.029	8	22
Mitotic G2-G2/M phases	0.034	20	110

Columns padj, Count and Size contain the Bonferroni adjusted p-values, the number of differentially expressed genes in the pathway and the total number of genes in the pathway, respectively.

Supplementary Table 4: Gene Ontology biological processes with significant overrepresentation of genes differentially expressed between 3D and 2D cultures of A549 cells

	Term	padj	Count	Size
GO:0032776	DNA methylation on cytosine	0.000	24	31
GO:0006335	DNA replication-dependent nucleosome assembly	0.000	24	32
GO:0051290	protein heterotetramerization	0.000	24	35
GO:0000183	chromatin silencing at rDNA	0.000	24	41
GO:0035574	histone H4-K20 demethylation	0.000	14	16
GO:0045653	negative regulation of megakaryocyte differentiation	0.000	14	18
GO:0006305	DNA alkylation	0.000	26	82
GO:0044728	DNA methylation or demethylation	0.000	26	95
GO:0031497	chromatin assembly	0.000	31	141
GO:0034080	CENP-A containing nucleosome assembly	0.000	17	42
GO:1901532	regulation of hematopoietic progenitor cell differentiation	0.000	17	42
GO:0071103	DNA conformation change	0.000	40	235
GO:0001568	blood vessel development	0.000	66	533
GO:0072358	cardiovascular system development	0.000	93	884
GO:0050878	regulation of body fluid levels	0.000	73	634
GO:0065004	protein-DNA complex assembly	0.000	32	174
GO:0034724	DNA replication-independent nucleosome organization	0.000	17	53
GO:0016577	histone demethylation	0.000	14	35
GO:0008214	protein dealkylation	0.000	14	37
GO:0051674	localization of cell	0.000	112	1229
GO:0045814	negative regulation of gene expression, epigenetic	0.000	27	148
GO:0070988	demethylation	0.000	17	63
GO:0000278	mitotic cell cycle	0.000	19	81
GO:0007264	small GTPase mediated signal transduction	0.000	85	893
GO:0060968	regulation of gene silencing	0.000	13	40
GO:0043044	ATP-dependent chromatin remodeling	0.001	17	73
GO:0051716	cellular response to stimulus	0.002	417	6663
GO:0051271	negative regulation of cellular component movement	0.009	29	209
GO:0051259	protein oligomerization	0.015	47	440
GO:0048864	stem cell development	0.022	23	151
GO:0051783	regulation of nuclear division	0.023	24	162
GO:0044700	single organism signaling	0.033	365	5855
GO:0045861	negative regulation of proteolysis	0.034	32	261
GO:0031100	organ regeneration	0.039	15	75
GO:0010951	negative regulation of endopeptidase activity	0.044	29	226

Columns padj, Count and Size contain the Bonferroni adjusted *p*-values, the number of differentially expressed genes in the term and the total number of genes in the term, respectively.

Supplementary Table 5: Gene Ontology biological processes with significant overrepresentation of genes differentially expressed between 3D and 2D cultures of Colo699 cells

	Term	padj	Count	Size
GO:0006323	DNA packaging	0.000	58	176
GO:0006335	DNA replication-dependent nucleosome assembly	0.000	26	32
GO:0071824	protein-DNA complex subunit organization	0.000	60	201
GO:0051290	protein heterotetramerization	0.000	26	35
GO:0032776	DNA methylation on cytosine	0.000	24	31
GO:0000183	chromatin silencing at rDNA	0.000	27	41
GO:0034080	CENP-A containing nucleosome assembly	0.000	25	42
GO:0034724	DNA replication-independent nucleosome organization	0.000	26	53
GO:0035574	histone H4-K20 demethylation	0.000	15	16
GO:0051276	chromosome organization	0.000	126	988
GO:0048285	organelle fission	0.000	83	549
GO:0045653	negative regulation of megakaryocyte differentiation	0.000	14	18
GO:0043044	ATP-dependent chromatin remodeling	0.000	26	73
GO:0071822	protein complex subunit organization	0.000	171	1629
GO:0044728	DNA methylation or demethylation	0.000	27	95
GO:0006305	DNA alkylation	0.000	25	82
GO:0045814	negative regulation of gene expression, epigenetic	0.000	34	148
GO:0006334	nucleosome assembly	0.000	26	93
GO:0016577	histone demethylation	0.000	16	35
GO:0051301	cell division	0.000	44	255
GO:0008214	protein dealkylation	0.000	16	37
GO:0000278	mitotic cell cycle	0.000	33	165
GO:0044770	cell cycle phase transition	0.000	50	312
GO:0070271	protein complex biogenesis	0.000	127	1201
GO:0007264	small GTPase mediated signal transduction	0.000	77	618
GO:1901532	regulation of hematopoietic progenitor cell differentiation	0.000	16	42
GO:0006333	chromatin assembly or disassembly	0.000	26	113
GO:0034622	cellular macromolecular complex assembly	0.000	91	790
GO:0070988	demethylation	0.000	18	63
GO:0022607	cellular component assembly	0.000	184	2082
GO:0098813	nuclear chromosome segregation	0.001	27	144
GO:0060968	regulation of gene silencing	0.003	13	40
GO:1903707	negative regulation of hemopoiesis	0.005	24	128
GO:0000086	G2/M transition of mitotic cell cycle	0.011	27	163
GO:0000723	telomere maintenance	0.013	15	60
GO:0051259	protein oligomerization	0.022	52	440

Columns padj, Count and Size contain the Bonferroni adjusted *p*-values, the number of differentially expressed genes in the term and the total number of genes in the term, respectively.

Supplementary Table 6: Reactome pathways with significant overrepresentation of genes differentially expressed between 5 day 3D and 5 day 2D cultures of A549 cells

Pathway name	padj	Count	Size
Packaging Of Telomere Ends	0.000	14	20
formation of the beta-catenin:TCF transactivating complex	0.000	19	44
HDMs demethylate histones	0.000	16	34
Condensation of Prophase Chromosomes	0.000	14	26
Amyloids	0.000	19	50
DNA Damage/Telomere Stress Induced Senescence	0.000	16	36
RMTs methylate histone arginines	0.000	15	32
Activated PKN1 stimulates transcription of AR (androgen receptor) regulated genes KLK2 and KLK3	0.000	15	33
DNA methylation	0.000	14	30
SIRT1 negatively regulates rRNA Expression	0.000	14	34
Meiotic synapsis	0.000	16	47
PRC2 methylates histones and DNA	0.000	14	39
TCF dependent signaling in response to WNT	0.001	31	168
Deposition of new CENPA-containing nucleosomes at the centromere	0.001	14	43
Nucleosome assembly	0.001	14	43

Columns padj, Count and Size contain the Bonferroni adjusted *p*-values, the number of differentially expressed genes in the pathway and the total number of genes in the pathway, respectively.

Supplementary Table 7: Gene Ontology biological processes with significant overrepresentation of genes differentially expressed between 5 day 3D and 5 day 2D cultures of A549 cells

	Term	padj	Count	Size
GO:0035574	histone H4-K20 demethylation	0.000	14	16
GO:0045653	negative regulation of megakaryocyte differentiation	0.000	14	18
GO:0016577	histone demethylation	0.000	16	35
GO:0006335	DNA replication-dependent nucleosome assembly	0.000	15	32
GO:0008214	protein dealkylation	0.000	16	37
GO:0032776	DNA methylation on cytosine	0.000	14	31
GO:2000145	regulation of cell motility	0.000	81	625
GO:0032502	developmental process	0.000	455	5551
GO:0070988	demethylation	0.000	19	63
GO:0051674	localization of cell	0.000	132	1229
GO:0051290	protein heterotetramerization	0.000	14	35
GO:0048869	cellular developmental process	0.000	331	3849
GO:0016265	death	0.000	182	1872
GO:0009888	tissue development	0.000	165	1680
GO:0001568	blood vessel development	0.000	71	553

Columns padj, Count and Size contain the Bonferroni adjusted *p*-values, the number of differentially expressed genes in the term and the total number of genes in the term, respectively.
